# Does cognitive impairment moderate the relationship between social isolation and anxiety? A 5-year longitudinal study of a nationally representative sample of community residing older adults

**DOI:** 10.1186/s12877-024-04685-z

**Published:** 2024-01-15

**Authors:** Yeji Hwang, Lauren Massimo, Subhash Aryal, Karen B. Hirschman, Pamela Z. Cacchione, Nancy A. Hodgson

**Affiliations:** 1 College of Nursing and Research Institute of Nursing Science, Seoul National University, 103 Daehak-ro, Jongno-gu, 03080 Seoul, Korea; 2 School of Nursing, University of Pennsylvania, Philadelphia, PA USA

**Keywords:** Social isolation, Loneliness, Anxiety, Cognitive impairment, Dementia

## Abstract

**Background:**

Anxiety is common in older adults and social isolation is one of the leading factors associated with their anxiety. However, what is unknown is how the relationship between social isolation and anxiety differs by cognitive status. Therefore, this study was conducted to (1) compare the level of social isolation and anxiety in older adults who developed probable dementia and mild cognitive impairment (MCI) to those who maintained normal cognitive function over 5 years; and (2) determine if cognitive impairment moderates the relationship between changes in social isolation and changes in anxiety over 5 years.

**Methods:**

A secondary data analysis was conducted using the National Social Life, Health, and Aging Project (NSHAP): Wave 2 (2010–2011) and Wave 3 (2015–2016). The participants were categorized into three groups: Participants who developed probable dementia over 5 years (4.3%), developed probable MCI (19.1%), or maintained normal cognitive function (76.6%). Weighted linear regression analyses with a group interaction were used to examine the moderating effect of cognitive impairment on the relationship between changes in social isolation and anxiety.

**Results:**

At the 5-year follow up, there were statistically significant differences in social isolation between the three groups (*p* = 0.043). Regression analyses showed that increased social isolation over time was related to increased anxiety over 5 years regardless of cognitive status after controlling for covariates (*p* = 0.017).

**Conclusions:**

The relationship between social isolation and anxiety was a universal phenomenon regardless of cognitive status. Tailored interventions targeting both people with or without cognitive impairment are needed to lessen social isolation and anxiety.

## Background

A lack of one’s social resources, such as companionship and social support, is known as social isolation [1]. When there is a discrepancy between desired and actual interpersonal relationships, people tend to perceive social isolation [2, 3]. Maintaining social relationships and a social support system is especially important in older adults as it is considered as part of successful aging [4, 5]. However, approximately a quarter of community-dwelling older adults living in the United States are socially isolated [6, 7]. Older adults experiencing social isolation may often show signs of negative responses, such as anxiety [8].

Anxiety is extremely common in older adults [9]. It is reported that up to 52.3% of older adults living at home experience anxiety [10]. According to previous literature, a leading factor associated with anxiety in older adults is feeling of isolation [8]. Findings from prior research suggests that poor social relationships with partners [11], poor social support [12], and fewer social activities [13] contribute to anxiety in older adults.

A high anxiety prevalence is also reported in older adults with cognitive impairment [14, 15]. Not only do people with cognitive impairment experience poor quality of life when anxiety is present [16], their family caregivers also experience more stress [16, 17]. Among many factors related to anxiety in older adults with cognitive impairment, social factors such as social isolation or social rejection are often associated with increased level of anxiety [18–20]. Recently, a study using national sample of American older adults also found that perceived social isolation is related to anxiety symptoms among people with cognitive impairment [21]. However, what is unknown is how the relationship between social isolation and anxiety differ by cognitive status.

Therefore, the aims of this study were: (1) To compare social isolation and anxiety among people who develop probable dementia and MCI and those who maintained normal cognition over a 5-year period; and (2) to determine if cognitive impairment moderates the relationship between changes in social isolation and changes in anxiety. We hypothesized that people who developed probable dementia or MCI would experience increased social isolation and anxiety at the 5-year follow up compared to those who maintained with normal cognitive function. We also hypothesized that there would be differences in the cognitive groups in terms of the strength of the relationship between changes in social isolation and changes in anxiety.

## Methods

### Participants

This study analyzed data collected from the National Social Life, Health, and Aging Project (NSHAP): Wave 2 (2010–2011) and Wave 3 (2015–2016). The NSHAP examined general health and social factors to understand the wellbeing of a nationally representative sample of older adults living at home in the United States [22, 23]. The NSHAP Wave 2 (2010–2011) included returning participants from the NSHAP Wave 1 (2005–2006) and a new cohort. The returning participants from the NSHAP Wave 1 were a nationally representative sample of adults whose ages were between 57 and 85 at the time of the Wave 1 interview. The new cohort was recruited from eligible, but non-interviewed respondents from the Wave 1, and cohabiting spouses or partners of the Wave 1 respondents or the Wave 1 non-interviewed respondents. In Wave 2, a total of 3,377 respondents participated in the study. There were 4,777 participants in the NSHAP Wave 3 (2015–2016) which included returning participants from the NSHAP Wave 2 (2010–2011) and a new cohort. Since this current study aimed to investigate cognitive changes from Wave 2 to Wave 3, only the returning cohort (i.e., people who both participated in Wave 2 and Wave 3) was used: The returning cohort had two time points: baseline (Wave 2) and 5 years after baseline (Wave 3). A total of 1,119 individuals who were at the age of 65 years old or older and had normal cognition at baseline (Wave 2) were included in the final sample. This study was approved by the Institutional Review Board at the authors’ institution.

### Measurements

#### Demographic variables

In this study, demographic variables including age, sex, education level, race, and employment status were used.

### Cognition

The NSHAP used the Montreal Cognitive Assessment (MoCA) Scale which is a well-known scale for cognitive assessment [24]. Suggested MoCA cutoff scores for cognitive impairment range between 22 and 26 [25–28], with community sample scores trending lower [27, 28]. Since the NSHAP is a community-based sample, the current study utilized the community MoCA cutoff scores [Normal: >22, probable MCI: 18–22, probable dementia: <18] [28, 29]. Participants whose cognitive function was normal at baseline were categorized into three groups based on their MoCA score at the 5-year follow up: (1) Participants who developed probable dementia (4.3%), (2) participants who developed probable MCI (19.1%), and (3) participants who maintained normal cognitive function (76.6%).

### Anxiety

The Hospital Anxiety and Depression Scale (HADS) was used to measure anxiety symptoms [30]. The HADS has 14 items, measuring anxiety symptoms (7 items) and depression (7 items). Only the anxiety measure (HADS-A) was used in this study. Each item is measured using a 4-point Likert scale, with a possible total score ranging from 0 to 21. The higher the HADS-A score indicates the more severe the anxiety. A higher HADS-A score indicates more severe anxiety. This scale has been frequently used in people with cognitive impairment [31–33].

The dependent variable of this study was *change in anxiety over 5 years*. In order to measure the change in anxiety scores between the baseline and the follow up, the HADS-A score at the baseline was subtracted from the HADS-A score at the 5-year follow up. A positive value indicated that the person had greater anxiety between the baseline and the 5-year follow up.

### Social isolation

The Perceived Social Isolation Scale is a 9 item scale examining loneliness and perceived lack of social support from family, friends, and spouse or current partner [1, 2]. Each item is scored using a 3-point Likert scale ranging from 1 to 3. Following the original scoring method of the scale [1, 2], each item was standardized with the mean of 0 and the standard deviation of 1, and the mean of the 9 standardized items indicated the level of social isolation. A higher score of social isolation indicates greater social isolation. Internal consistency of the scale was acceptable when it was developed [2].

To measure the change in social isolation between the baseline and the 5-year follow up, we followed the method based on the scoring method of the scale [1, 2]. For each 9 items, the baseline value was subtracted from corresponding follow up value, with a positive score indicating that a person had greater social isolation between the baseline and the 5-year follow up. Next, the change scores of each item were standardized, and the mean value of the 9 standardized change scores was used to present the change in social isolation between the baseline and the 5-year follow up. Greater overall values indicated that the person experienced greater social isolation between the baseline and the 5-year follow up. The reliability of the Perceived Social Isolation Scale was acceptable in this study with Cronbach’s alpha value of 0.7.

Because the scoring method suggests using the mean score [1, 2] and our study examines the change score between the two time points, it is possible that the change scores of each item may not have same direction of change (e.g., While the change score of Item #1 may hold positive values, the change score of Item #2 may hold negative values.). Averaging the 9 items with different direction of change may not precisely represent the overall trend of the 9 items. Therefore, in this study, in addition to the mean value of the 9 standardized change scores, we planned to use individual standardized change scores of the 9 items.

### Covariates

Covariates included depressive symptoms and basic activities of daily living. Covariates were selected based on their known relationships to anxiety [34–37]. Depressive symptoms were measured with Center for Epidemiologic Studies Depression Scale (CES-D) with higher scores indicating greater depressive symptoms [38]. The total score of basic activities of daily living was included as a covariate in this study. Basic activities of daily living were measured with 7-item scale with the higher scores demonstrating better functional independence.

### Data analysis

Both the NSHAP Wave 2 and Wave 3 provide person-level weight which adjusts for differential probabilities of selection and non-response [23]. In order to ensure representativeness, this study used the weights to all statistical analysis.

A descriptive data analysis was conducted by calculating the means, standard deviations, and percentages to illustrate the characteristics of the participants. Adjusted Wald tests were used to compare the level of social isolation and anxiety between three groups: Individuals who developed probable dementia or MCI and those who did not (defined by MoCA cutoffs described above), from the initial baseline assessment to the 5-year follow-up. To examine the moderating effect of cognitive impairment on the relationship between changes in social isolation and anxiety between the baseline and the 5-year follow up, a weighted linear regression model with an interaction term between cognitive group and changes in social isolation was used.

Before performing linear regression analysis, correlation coefficients between the independent variable, covariates, and the dependent variable were calculated. Only variables with statistically significant correlation (*p* < 0.05) among them were included in the final regression model. We used listwise deletion and only the complete cases were used for the final regression model. We evaluated the regression assumptions using residuals to check any violations. All analyses were performed using Stata BE 17 [39]. The level of statistical significance was set at *p* < 0.05.

## Results

Table 1 describes the weighted characteristics of the study participants. The weighted mean age was 71.8 years (SD = 5.8). Approximately 45.1% were male, and 54.9% were female. Among the 1,119 participants who were cognitively intact at the baseline, 76.6% of the participants remained cognitively intact at the 5-year follow up; 19.1% of the participants were categorized as having probable MCI; and 4.3% of the participants were categorized as having probable dementia.

**Table 1:** Weighted sample demographic and characteristics (*n* = 1,119)^*^

	M ± SD or Percent(%)
**Baseline Characteristics**	
Age	71.831 ± 5.779
Sex	
Male	45.134
Female	54.866
Education	
Less than High School	5.737
High School Diploma/Equivalency	23.792
Vocational Certificate or Some College	34.211
College degree or higher	36.260
Race	
White / Caucasian	92.071
Black / African American	4.976
American Indian / Alaskan Native / Asian / Pacific Islander	1.763
Other	1.189
Employment Status	
Employed	26.708
Not Employed	73.292
Pain	
Pain in Past 4 Weeks	67.096
No Pain in Past 4 Weeks	32.904
Depression	3.592 ± 3.712
Basic Activities of Daily Living	6.486 ± 1.127
**Dependent variable**	
Change in Anxiety	-0.113 ± 0.000
**Independent variable**	
Changes in Social Isolation	0.009 ± 0.497
Changes in Cognition	1.247 ± 3.095
Maintained Normal Cognition	76.589
New Development of Mild Cognitive Impairment	19.141
New Development of Dementia	4.270

^*^The unweighted total number of participants were 1,119

Note: Changes in Anxiety indicates anxiety scores in Wave 3 minus anxiety scores in Wave 2, and positive scores indicate increased anxiety over 5 years; Changes in Social Isolation Scales indicates social isolation scores in Wave 3 minus social isolation scores in Wave 2 and positive scores indicate increased social isolation; Changes in Cognition scores indicate cognition scores in Wave 2 minus cognition scores in Wave 3 and positive scores indicate increased cognition impairment

The weighted means of social isolation and anxiety were compared among the three cognitive groups. At the 5-year follow up, there were statistically significant differences in social isolation between the three groups (F = 3.37, *p* = 0.043); whereby people who developed probable dementia and MCI tended to have higher ratings of social isolation. However, there were no differences between the anxiety scores of the three groups (F = 1.67, *p* = 0.199) (Table 2).

**Table 2:** Weighted Means of Social Isolation and Anxiety among People who Developed Cognitive Impairment and People who Remained Cognitively Intact

Variables	Maintained Normal Cognition (n = 837)	Developed Mild Cognitive Impairment (n = 226)	Developed Dementia (n = 56)	F	*p*
Social Isolation in Wave 3	-0.039	0.066	0.119	3.37	**0.043**
Anxiety in Wave 3	4.027	4.394	4.690	1.67	0.199

Note: Montreal Cognitive Assessment (MoCA) cutoff score of 22 were used for the cognitive group. People who were cognitively intact in Wave 2 were followed up in Wave 3 and categorized into three groups; a group who maintained normal cognition, a group who developed probable MCI, and a group who developed probable dementia

The second aim of the study was to determine if cognitive impairment moderates the relationship between changes in social isolation and changes in anxiety. We first conducted a weighted univariate regression analysis to determine if an increase in the social isolation scale was related to increased anxiety over years. Findings indicated that changes in social isolation scale were not related to changes in anxiety (B = 0.463, SE = 0.340, t = 1.36, *p* = 0.179). We evaluated the social isolation scale’s change score for each of the 9 items to examine if the mean value represented the overall trend of the summary score. As shown in Table 3, while the scores for item 1 and item 4 of the social isolation scale decreased 5-years later, the scores of the remaining items increased 5-years later. When averaging the 9 items of the social isolation scale, it is likely that the mean value does not represent the overall trend of the 9 items in the social isolation scale. Therefore, we decided to analyze each item separately to examine which aspects of social isolation was related to changes in anxiety.

**Table 3:** Changes in social isolation scale from wave 2 to wave 3 (*n* = 1,119)

Perceived Social Isolation Score (Wave 3-Wave 2)	n	Mean	SD
Loneliness (1 = hardly ever or never, 2 = some of the time, 3 = often)			
1. How often do you feel that you lack companionship?	938	-0.026	0.640
2. How often do you feel left out?	938	0.002	0.554
3. How often do you feel socially isolated from others?	933	0.009	0.512
Perceived Social Support (1 = often, 2 = some of the time, 3 = hardly ever or never)			
4. How often can you open up to members of your family?	1004	-0.020	0.833
5. How often can you rely on members of your family?	1008	0.074	0.717
6. How often can you open up to your friends?	1022	0.073	0.775
7. How often can you rely on your friends?	1020	0.132	0.802
8. How often can you open up to your spouse or partner?	750	0.073	0.586
9. How often can you rely on your spouse or partner?	747	0.044	0.471

Note: Positive mean score indicates increased social isolation over 5 years

We conducted weighted univariate regression analyses on changes in anxiety using the 9 items of the social isolation scale. Among the 9 items, changes in one item showed marginal significance and another item showed statistical significance at alpha = 0.05. Increased feeling of being left out was marginally related to increased anxiety (B = 0.613, SE = 0.310, t = 1.98, *p* = 0.053), and increased feeling of social isolation was significantly related to increased anxiety (B = 0.750, SE = 0.262, t = 2.86, *p* = 0.006). Based on these results, we decided to separately use changes of these 2 items as changes in social isolation, as they were related to the dependent variable, changes in anxiety.

Before we conducted the final regression analysis, we examined whether baseline characteristics listed in Table 1 were related to changes in anxiety using correlation coefficients test. Baseline depressive symptoms (*r*=-0.110, *p* = 0.041) and greater independence in basic activities of daily living at baseline (*r*=-0.091, *p* = 0.037) were related to decreased anxiety. Thus, both covariates were included in the final multivariable regression modeling.

The weighted multivariable linear regression analysis was conducted on changes in anxiety. We graphically evaluated the regression assumptions using raw residuals and we did not identify any violation of regression assumptions. In addition, we have a large sample size, and our analysis is robust to minor assumption deviations. Multicollinearity between the independent variables and covariates was checked using the VIF, and all values were under 10, indicating no multicollinearity. Table 4 shows the results of the weighted multivariable linear regression analysis on changes in anxiety. After controlling for covariates, increased feeling of social isolation over 5 years was related to increased feeing of anxiety over 5 years (B = 0.725, SE = 0.293, t = 2.47, *p* = 0.017). There were no statistically significant interactions for the cognitive groups.

**Table 4:** Weighted Multivariable Linear Regression on Changes in Anxiety (*n* = 920)

		B	SE	t	*p*
*Independent variable*	Changes in PSI: Loneliness Item 2: Feeling Left Out	0.523	0.307	1.71	0.094
	Item 3: Feeling Socially Isolated	0.725	0.293	2.47	**0.017**
	Cognition				
	Maintained Normal Cognition	reference			
	Development of Probable MCI	-0.284	0.333	-0.85	0.397
	Development of Probable Dementia	-0.187	0.813	-0.23	0.819
	Interaction Terms (×)				
	Item 2 × Development of Probable MCI	-0.235	0.547	-0.43	0.669
	Item 2 × Development of Probable Dementia	0.327	0.953	-0.34	0.733
	Item 3 × Development of Probable MCI	-1.26	0.862	-1.46	0.150
	Item 3 × Development of Probable Dementia	-1.04	2.16	-0.49	0.629
*Covariates*	Basic Activities of Daily Living	-0.388	0.163	-2.38	0.021
	Depression	-0.133	0.056	-2.38	0.021
	Constant	2.931	1.172	2.50	0.016
	R^2^ = 0.05, F = 2.81, *p* = 0.0094				

Note: Montreal Cognitive Assessment (MoCA) cutoff score of 22 were used for the cognitive group. People who were cognitively intact in Wave 2 and developed cognitive impairment in Wave 3 were categorized as ‘New Development of Probable Mild Cognitive Impairment (MCI) (n = 226)’ and ‘New Development of Probable Dementia (n = 56).’ People who were cognitively intact in Wave 2 and again in Wave 3 were categorized as ‘Maintained Normal Cognition (n = 837).’; ×=interaction; PSI = Perceived Social Isolation

## Discussion

In this study, we compared social isolation and anxiety among people who developed probable dementia, who developed probable MCI, and those who maintained normal cognition over a 5-year period. As hypothesized, people who developed probable cognitive impairment experienced greater social isolation compared to people who maintained normal cognition. This result aligns with a previous study which found that people living with dementia experience more social isolation compared to older adults with normal cognition [40]. Loss of cognitive function can make it difficult for people to engage in social activities and this can exclude them from society. However, there was no difference in anxiety between people who developed probable dementia or MCI and people who maintained normal cognition 5 years later. This might have been due to individual personality or different coping strategies that this study was unable to capture. Future studies should consider these potentially important covariates.

In our study, we also examined if there was a moderating effect of cognitive impairment on the relationship between social isolation and anxiety. While changes in social isolation were related to increased anxiety, there were no observable differences between cognitive groups, indicating that people with cognitive impairment can feel loneliness and anxiety in the same manner that people without cognitive impairment perceive. Tailored interventions that can support older adults to feel less isolation should be developed and tested.

There are limitations of this study. First, there was a disparity in group sizes with the group of people who remained intact cognition (76.6%) being notably greater than the group of people who developed probable MCI (19.1%) or probable dementia (4.3%). Although it is natural that only small percentage of people develop cognitive impairment over time, there may be a skewed data representation due to the uneven group sizes. Therefore, conclusions drawn from these comparisons should be approached with caution.

Next, only two time points for social isolation and anxiety were available: The baseline and the 5-year follow-up. If there were additional information available between the 5 years, it might have generated richer information. In our study, the R^2^ value in our final regression model was 0.05. This study was a secondary data analysis of the NSHAP, which includes a nationally representative sample of older adults in the United States. Although our subsample is not representative of all American older adults, it is representative of American older adults who were 65 years old or older and who had normal cognition at baseline. Considering that this study utilized a subsample of a nationally representative sample of older adults in the United States, the estimates may be important in the population.

There are several limitations about measurements. It is a limitation that the MoCA does not serve as a comprehensive diagnostic tool for dementia or MCI, and there are a variety of cutoff scores used to screen for dementia or MCI. Therefore, cautions should be used when interpreting the results of the study. In this study, it is a limitation that the social isolation scale was not used as whole due to the scoring method of the scale using the mean score. Additionally, it is important to note that there are various ways of defining and measuring social isolation. Results might have been different if social isolation was measured differently.

Finally, since we used secondary data analyses, information on individual coping strategies or duration since cognitive decline was not available in the study which might have been important confounding variables to changes in anxiety over time. Therefore, future longitudinal studies including additional information are needed to better understand the relationship between cognition impairment, social isolation, and psychological outcomes.

## Conclusions

This is one of the few studies that has focused on how social isolation is related to anxiety in people with and without cognitive impairment. This study showed that people who developed probable cognitive impairment experienced higher social isolation compared to people who maintained intact cognition, and increased social isolation was related to increased anxiety regardless of cognition status. Health care professionals should pay close attention in assessing social needs of older adults, and more research efforts are needed to improve social isolation among these people.

## Data Availability

The datasets generated and/or analyzed during the current study are available in the National Archive of Computerized Data on Aging repository, 10.3886/ICPSR34921.v4 and 10.3886/ICPSR36873.v4.

## Abbreviations

MCI: Mild cognitive impairment
NSHAP: National Social Life, Health, and Aging Project
MoCA: Montreal Cognitive Assessment
HADS: Hospital Anxiety and Depression Scale
SD: Standard Deviation
